# Brain Region-specific Accumulation of Amyloidosis-associated Proteins in Postmortem Brain Tissues of Alzheimer’s Disease Patients

**DOI:** 10.21203/rs.3.rs-6649354/v1

**Published:** 2025-06-03

**Authors:** Wangchen Tsering, Jennifer L. Philips, Todd E. Golde, Jonathan A. Villareal, Stefan Prokop

**Affiliations:** University of Florida; University of Florida; Emory University; University of Florida; University of Florida

**Keywords:** Alzheimer’s disease, Aβ plaques, Amyloidosis-associated proteins, neuritic plaques, dystrophic neurites

## Abstract

Numerous extracellular matrix (ECM) proteins, referred to as the matrisome, are increased in Alzheimer’s disease (AD). We recently demonstrated that many of these proteins colocalize with Aβ plaques and cerebral amyloid angiopathy (CAA), and some are present in dystrophic cellular processes within and around plaques. However, their precise roles in AD pathogenesis and their spatial and temporal distribution in postmortem brain tissue remain incompletely understood. Here, we performed a comprehensive immunohistochemistry analysis on postmortem brain samples spanning the spectrum of AD neuropathological change (ADNC: low, intermediate, and high). We assessed the accumulation of five matrisome proteins (MDK, SPOCK3, COL25aA1, SDC4, and EGFL8) across four brain regions differentially affected in AD (occipital cortex, hippocampus, striatum, and cerebellum), and examined their association with Aβ plaques, CAA, tau neurites, and neurofibrillary tangles (NFT). MDK in plaques increased consistently with ADNC severity across all regions. In contrast, SPOCK3, COL25A1, EGFL8, and SDC4 showed marked accumulation only in the occipital cortex and hippocampus, with sparse presence in the striatum and absence in the cerebellum. Notably, SPOCK3 exhibited pronounced regional specificity, with significantly higher levels in the hippocampus than in other areas. Patterns of plaque staining and degree of colocalization indicate that select matrisome proteins associate with either distinct types of Aβ deposits (e.g, fibrillar and neuritic versus diffuse plaques), while others may correlate more closely with tau pathology and/or dystrophic processes around plaques. Overall, our findings reveal region- and pathology-specific patterns of these matrisome protein accumulation during AD progression. These proteins represent intriguing biomarkers of AD and based on modeling studies represent potential therapeutic targets.

## Introduction

The extracellular matrix (ECM) is the non-cellular component that provides structural support and regulates cell adhesion, differentiation, and migration[21, 29, 37, 50]. The ECM is largely composed of collagen, proteoglycans, and glycoproteins[50]. Heparan sulfate proteoglycans (HSPGs) and chondroitin sulfate proteoglycans (CSPGs), components of the ECM, have been historically shown to be implicated in AD[16, 61, 62, 71]. Recent proteomic studies have shown that ECM and ECM-related proteins, collectively known as the “matrisome”, are significantly altered in AD patients [1, 34, 35, 41]. A large-scale study of over 8600 proteins from nearly 1000 postmortem brain samples demonstrated that matrisome proteins were not only enriched in AD compared to healthy controls but also correlated well with progression of AD pathology and cognitive decline [34]. Interestingly, a study comparing proteomic and transcriptomic data from the same brain samples revealed that while matrisome proteins are more abundant in AD samples compared to healthy controls, no changes were observed in the levels of encoding RNA[34]. This disconnect between RNA levels and protein abundance suggests that these molecules are accumulating during progression of AD. Apart from depositing in the brain parenchyma, matrisome proteins are also detected in CSF and plasma samples of AD patients[35].

Some matrisome proteins, such as APOE, APP, and CLU have previously been shown to modulate Aβ deposition and tau aggregation in AD [15, 19, 27]. A whole-genome sequencing (WGS) study of APOE4 carriers without dementia identified that the rs140926439 variant in fibronectin 1, an ECM-related gene, is protective against AD and can delay the age onset of the disease [7]. Similarly, rare variants in Reelin and APOE, both of which are ECM related genes, can stave off cognitive decline for many years in individuals with autosomal dominant AD [46, 51]. These studies suggest that matrisome proteins play an important role in the cascade of events leading to dementia in AD and could be potential therapeutic targets.

We recently demonstrated that many matrisome proteins are upregulated in transgenic animal models of AD [41, 74] and validated these findings using newly generated antibodies against matrisome proteins on human brain tissues[41]. We showed that these matrisome proteins accumulate in Aβ plaques, cerebral amyloid angiopathy (CAA), and dystrophic neurites (DN) in AD patient brain samples [41]. Overexpression of two matrisome proteins, PTN and MDK in the CRND8 mouse model exacerbated the deposition of Aβ in plaques and CAA [41], suggesting that some matrisome proteins are not simply bystanders but can actively modify the course of Aβ pathology. Quantifying the accumulation of these Amyloidosis-associated proteins (AAP) in postmortem brain samples, we furthermore demonstrated that accumulation of some AAP increased in the frontal cortex of AD patients following the progression of ADNC from low over intermediate to high, mimicking the progression of non-neuritic plaques (non-NP) we had previously reported[68]. Other AAP only showed significant accumulation in cases with intermediate and high ADNC, following the trajectory of neuritic plaque (NP) formation[68].

Given the association of some AAP with different phases of Aβ deposition and maturation, we speculate that individual AAP may serve as biomarkers that reflect the transition from non-NP to NP or may even be triggers of this pathophysiological process.

To further home in on this hypothesis, we analyzed the temporal and spatial accumulation of select AAP during the progression of ADNC and correlation of AAP accumulation with non-NP, NP, CAA, DN, and NFT in different disease relevant brain regions across the spectrum of ADNC. To this end, we selected secreted factors (MDK, EGFL8), a proteoglycan (SPOCK3), an ECM-affiliated protein (SDC4), and a collagen component (COL25A1)[50] for our analyses. These matrisome proteins are some of the most highly differentially regulated proteins in AD[2, 34]. MDK is neurite promoting growth factor [54] and is expressed by fetal astrocytes in human brains [58]. SPOCK3 is a calcium-binding proteoglycan and a member of the SPARC/osteonectin family of glycoprotein, expressed mostly by oligodendrocytes and neurons in the adult brain [12, 23]. Syndecan-4 (SDC4) is a transmembrane proteoglycan that functions as a receptor between cells and the ECM, regulating cell migration and cytoskeleton organization[55]. It is mostly expressed by mature astrocytes in human brains[75]. COL25A1 is a membrane-associated collagen expressed by neurons which was previously described as a component of Aβ plaques in AD[20, 25]. EGFL8 (epidermal growth factor-like protein 8) is a growth factor prominently expressed by endothelial cells and is involved with cell proliferation[43] and linked with white matter hyperintensities[48].

We found that all the examined matrisome proteins accumulate during the progression of ADNC (i.e. Low ADNC to High ADNC) in the occipital cortex and hippocampus. However, only MDK showed significant accumulation in the striatum, where almost exclusively non-NP are found during the progression of ADNC. MDK accumulation was observed in all brain regions, while COL25A1, SPOCK3, SDC4, and EGFL8 accumulation was minimal in the mostly diffuse (non-NP) Aβ pathology in the striatum and completely absent in the scattered diffuse Aβ deposits in the cerebellum. Notably, SPOCK3 showed a region-specific accumulation pattern with significant accumulation in the hippocampus compared to other brain regions. SPOCK3 and SDC4 also co-localized with a subset of tau pathology, indicating possible connection of these two proteins in Aβ associated tau accumulation and spread.

## Methods

### Patient Samples

Human brain samples were provided by the University of Florida Human Brain and Tissue Bank (HBTB). Autopsy cases were grouped into “Low AD” (n = 6), “Intermediate AD” (n = 6), and “High AD” (n = 6) based on the NIA-AA guideline for the neuropathological assessment. Information related to protocol approval, ADNC classification, cognitive score conversion etc. are detailed in our previous articles [5, 68]. Neuropathological and case demographic details of the samples used in this study are shown in Table 1 and Supplementary Table 2. Four brain regions (occipital cortex, hippocampus, striatum, and cerebellum) from each sample were stained and analyzed.

#### Immunohistochemistry (IHC) Staining.

8-μm thick-sections of formalin-fixed, paraffin-embedded (FFPE) postmortem brain tissues were deparaffinized by immersing them in xylene twice, each for 5 minutes, followed by rehydrating in ethanol series (100%, 100%, 90%, 70%) for 1 minute per step. For heat-induced epitope retrieval (HIER), sections were incubated in 0.1M Tris and 0.05% Tween at high pressure in a pressure cooker (Tintoretriever, Bio SB) for 15 minutes, followed by incubation in a 30% H_2_O_2_ solution (diluted with PBS) and 10% Triton-X for 20 minutes to quench endogenous peroxidase. The sections were rinsed multiple times with tap water and then washed in 0.1M Tris for at least 5 minutes. Blocking steps included incubation in in normal horse serum for 20 minutes, followed by 2% FBS/0.1 M Tris, (pH 7.6) for 5 minutes. Next, primary antibody, diluted in blocking buffer was applied on the section and incubated overnight at 4°C. Antibody details are provided in Supplementary Table 3 and can also find in [41]

Following day, sections were rinsed in 0.1M Tris and blocked again in 2% FBS/0.1 M Tris, (pH 7.6) for 5 minutes before incubating with the secondary antibody (HRP-conjugated ImmPRESS Polymer Reagent, Vector Labs) for 30 minutes in room temperature. After a quick wash in 0.1M Tris, 3,3’-diaminobenzidin (DAB, Vector Lab SK-410) was applied for 1–5 minutes to visualize the staining and then sections were counterstained with hematoxylin (Mayer’s version, Sigma Aldrich) for 1 minute. Next, sections were rinsed in tap water, and then dehydrated through ethanol series (70%, 90%, 100%, 100%) for 1 minute each per step, followed by washing in xylene (2 x 5 mins). Finally, sections were cover-slipped using Cytoseal 60 (Thermo Fisher) mounting media and dried overnight.

#### Immunofluorescent (IF) Staining.

For immunofluorescence, 8-μm thick FFPE postmortem brain tissues were used. The deparaffinization, antigen retrieval, and primary antibody incubation steps followed the same protocol as described above for IHC. Following overnight incubation with the primary antibodies, slides were washed and immersed in 2% FBS/0.1 M Tris, (pH 7.6) for 5 minutes. Subsequently, a fluorophore-conjugated anti-mouse secondary antibody was mixed with anti-rabbit ImmPRESS Polymer reagent (Vector Labs) and applied to the slides for 1 hour. After incubation, slides were rinsed in 0.1M Tris and blocked with Multiplex TSA Buffer (ACD Bio, ref# 322809) for 5 minutes, followed by incubation with Opal 570 Reagent (Akoya Biosciences, product # OP-001003) diluted 1:1000 in Multiplex TSA buffer. Finally, slides were washed and mounted with a coverslip using mounting media containing DAPI.

### Data Analysis

IHC-stained brain slides were scanned using an Aperio AT2 slide scanner (Leica Biosystems) at 40x magnification. Scanned slides were both automatically (using a script) and manually annotated in Qupath, open-source digital pathology software[4]. Grey matter regions of occipital cortex, hippocampus, basal ganglia (striatum), and cerebellum were annotated. For quantification of pathology in annotated brain regions, script for the “Positive Pixel Count” was used in Qupath to measure the percent of area covered by pathology. The threshold for Positive Pixel Count includes “Downsample factor” of 4.0, “Gaussian sigma” of 2–4 um, Hemotoxylin threshold of 1 OD unit, and DAB threshold of 0.2–0.4 um. Gaussian sigma and DAB threshold parameter were adjusted for individual slides and for different antibodies. Details of Qupath scripts (Positive Pixel Count, Automatic annotation) were in previously published manuscripts from our laboratory [5].

For immunofluorescent colocalization analysis, confocal microscopy (Nikon CSY-W1 SoRA) was used to acquire 3–6 fields of view (FOV) images per sample from the hippocampus and fusiform gyrus (FG) + inferior temporal gyrus (ITG). Images were captured using the same channel settings, including laser power and exposure time. To measure the colocalization between matrisome proteins and Aβ plaques, the BIOP-JACoP plugin [8] in ImageJ was used to obtain Pearson’s correlation coefficient (PCC) and Manders’ overlap coefficients (MOC). PCC measures the linear correlation between the intensity values of two fluorescent channels. A PCC of 1 indicates perfect positive correlation, o indicates no correlation, and −1 indicates perfect negative correlation. MOC measures the fraction of signal from one channel that spatially overlaps with signal from the other. A MOC of 0 indicates no overlap, while a value of 1 indicates complete overlap. Images were thresholded using “Otsu” automatic thresholding method before colocalization analysis to offset background signal and minimizes intra-class variance. Workflow of the BIOP-JACoP analysis is in supplementary Fig. 1.

### Statistics

All the statistical analyses were performed in GraphPad Prism (Version 10.2.3). One-way analysis of variance (ANOVA) with Tukey’s multiple comparison test was used to examine the mean differences between groups. All data are presented as standard deviation (SD) of the mean. No outlier test was conducted to exclude any data points.

## Results

### Accumulation of AAP during progression of ADNC in occipital cortex and hippocampus

To investigate the accumulation and brain region-specific distribution of select AAP (SPOCK3, MDK, COL25A1, EGFL8, SDC4) during the progression of ADNC, postmortem brain tissues representing different ADNC stages (low ADNC, n = 6; intermediate ADNC, n = 6; high ADNC, n = 6) were used in this study. ADNC staging was based on the NIH-AA guideline[28, 49] and has been described previously for the cohort used in this study[5, 68]. We quantified the accumulation of SPOCK3, MDK, COL25A1, EGFL8, and SDC4 in four disease relevant brain regions (occipital cortex, hippocampus, striatum, and cerebellum). Antibodies against matrisome proteins were characterized and validated previously [41].

**Figure 1:**
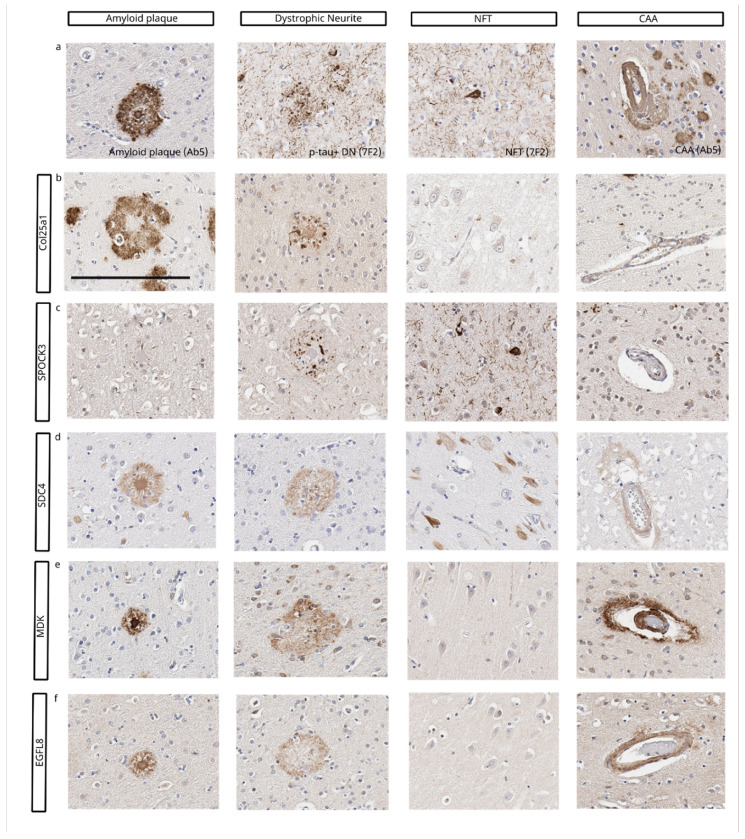
Matrisome proteins stain different pathological hallmarks of AD. a) Pathological hallmarks of AD – Aβ plaques and CAA stained with an anti- Aβ antibody (Ab5), and DN and NFT stained with an anti-phospho tau antibody (7F2) b) COL25A1 predominently stained Aβ plaques, occassionally stained DN and CAA, and did not stain NFT. c) SPOCK3 exclusively stained DN and neuronal tau aggregates. d) SDC4 predominantly stained Aβ plaques and CAA, and occasionally stained neuronal tau aggregates in hippocampus. e) MDK stained Aβ plaques and CAA. f) EGFL8 stained Aβ plaques and CAA. Scale bar = 200 μm

Qualitatively, matrisome proteins stain different pathological features of AD pathology. MDK stains parenchymal Aβ deposits and CAA (Fig. 1). COL25A1 staining colocalizes mostly with Aβ plaques and also stains sparse dystrophic neurites (Fig. 1). SPOCK3 antibodies stain mostly dystrophic neurites and some neuronal tau aggregates (Fig. 1). SDC4 antibodies mostly label Aβ plaques and minimally stain neuronal tau aggregates and CAA (Fig. 1). EGFL8 antibodies stain parenchymal Aβ plaques and CAA (Fig. 1).

**Figure 2:**
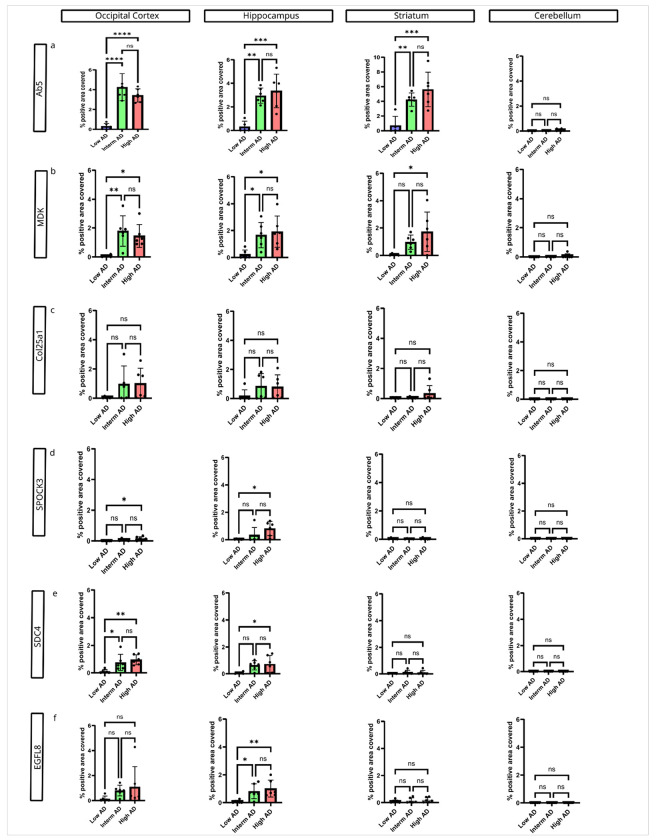
Accumulation of matrisome proteins during the progression of ADNC. A) Aβ plaque burden, as measured by percentage of Ab5-positive area, significantly increased from Low AD (n=6) to Interm AD (n=6) and High AD (n=6) across all brain regions examined except the cerebellum. In the cerebellum, a few diffuse plaques were observed in cases with High ADNC, but no statistically significant difference was detected by pixel positivity analysis. B) MDK pathology burden significantly increased from Low AD to High AD in all brain regions except the cerebellum. C) No significant differences in COL25A1 burden were observed across brain regions; however, a trend toward increased burden from Low AD to High AD was noted in the occipital cortex and hippocampus. D) SPOCK3 pathology burden significantly increased from Low AD to High AD in the occipital cortex and hippocampus, with no differences in the striatum or cerebellum. E) SDC4 pathology was more abundant in High AD cases compared to Low AD cases in the occipital cortex and hippocampus with no differences observed in the striatum or cerebellum. F) EGFL8 burden was significantly higher in Interm AD and High AD compared to Low AD in the hippocampus, with no significant differences in other brain regions. Sample size: Low AD (n=6), Interm AD (n=6) and High AD (n=6).

To quantify our finding in relation to Aβ deposition, we first assessed the accumulation of Aβ plaques using the anti-Aβ antibody Ab5[42]. As expected, we observed that Aβ plaques significantly increased with progression of ADNC from low over intermediate to high ADNC in all brain regions examined except for the cerebellum (Fig. 2). In the cerebellum, cases with high ADNC showed variable diffuse Aβ deposits, but the results did not reach statistical significance when Aβ deposition was quantified by total pixel count across cases (Fig. 2).

Similar to the trajectory observed for Aβ plaques MDK burden significantly increased from low over intermediate to high ADNC in the hippocampus, occipital cortex and striatum (Fig. 2). There were no differences in MDK load between ADNC groups in the cerebellum. The general trajectory of MDK accumulation during ADNC progression is comparable to the deposition of Aβ plaques as assessed by Ab5 staining.

COL25A1 accumulation trends towards an increase from low to high ADNC in the hippocampus and occipital cortex, but these results did not reach statistical significance (Fig. 2).

The burden for both SPOCK3 and SDC4 increased from low to high ADNC in the hippocampus and occipital cortex (Fig. 2). Interestingly, we did not observe significant differences between any ADNC groups in the striatum and cerebellum for SPOCK3 and SDC4 accumulation.

EGFL8 pathology load significantly increased from low over intermediate to high ADNC in the hippocampus while a trend towards increased deposition was observed in the occipital cortex. Like SPOCK3 and SDC4, EGFL8 burden was not changed in the striatum and cerebellum during ADNC progression (Fig. 2).

In summary, all AAP examined in this study show increased deposition during progression of ADNC from low to high ADNC in the occipital cortex and hippocampus following the trajectory of NP accumulation, while this trajectory of deposition is only observed for MDK in the striatum and cerebellum, where diffuse Aβ deposits predominate.

### Brain region-specific differences in accumulation of matrisome proteins

**Figure 3:**
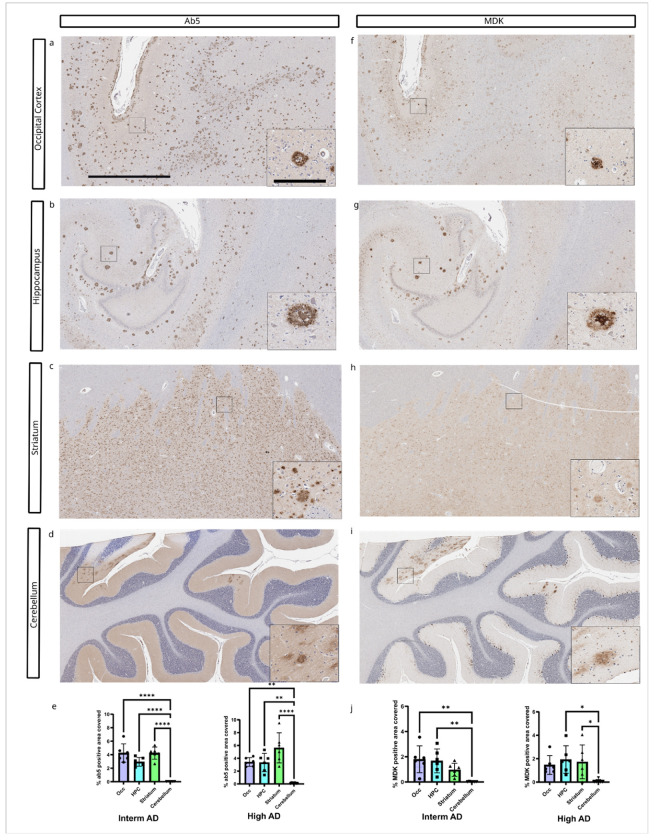
Brain region-specific accumulation of Aβ plaques and MDK. A-D) Aβ plaque pathology in different brain regions from consecutive tissues of a high AD case (sample 16). E) Aβ plaque pathology was abundant in the occipital cortex, hippocampus, and striatum, and was significantly greater compared to the cerebellum in Interm AD and High AD cases. F-J) MDK burden was elevated in the occipital cortex, hippocampus, and striatum. In Interm AD cases, MDK load was significantly higher in the occipital cortex and hippocampus compared to the cerebellum. In High AD cases, MDK burden was significantly greater in the hippocampus and striatum compared to the cerebellum. Sample size: Interm AD (n=6) and High AD (n=6). Scale bar = 2 mm for the main figure and 200 μm for insets.

Aβ plaques and tau pathology propagate in a brain region-specific manner. According to Thal et al.[66], Aβ plaque deposition initiates in neocortical regions, followed by the hippocampus and basal ganglia and eventually spreads to the brainstem and cerebellum[66]. Since SPOCK3, MDK, SDC4, and COL25A1 co-deposit with Aβ plaques and we observed differences in the accumulation of these matrisome proteins in different brain regions, we compared the relative deposition of AAP between occipital cortex, hippocampus, striatum and cerebellum compared to Aβ plaques. Aβ plaque pathology is extensive in the occipital cortex, hippocampus, and striatum in cases with intermediate and high ADNC (Fig. 3). Similar to the distribution of Aβ plaques, the MDK burden was comparable between the occipital cortex, hippocampus, and striatum in cases with intermediate and high ADNC (Fig. 3). Aβ plaque and MDK pathology load assessed by percent area covered are significantly higher in the occipital cortex, hippocampus and striatum compared to the cerebellum. In the cerebellum, both the Aβ antibody and the MDK antibody exclusively labelled diffuse Aβ deposits.

**Figure 4:**
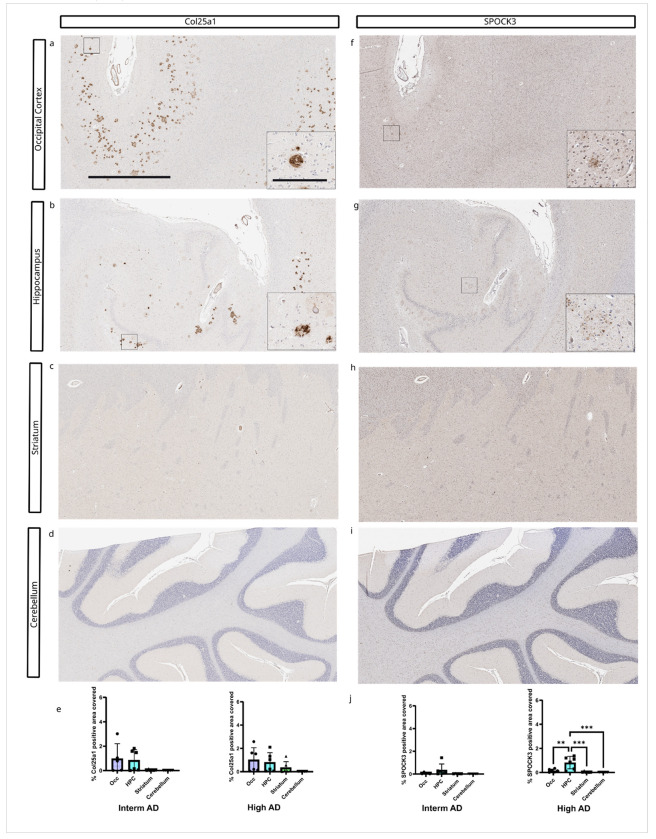
Brain region-specific accumulation of COL25A1 and SPOCK3. A-D) COL25A1 staining in different brain regions from consecutive sections of a high AD case (sample 16). E) COL25A1 pathology burden did not significantly differ across all examined brain regions. F-J) SPOCK3 pathology in different brain regions. J) In cases with Interm ADNC, there were no significant differences in SPOCK3 burden between brain regions. However, the SPOCK3 burden was significantly higher in the hippocampus compared to all other brain regions. Sample size: Interm AD (n=6) and High AD (n=6). Scale bar = 2 mm for the main figure and 200 μm for insets.

We did not observe any significant differences in percent area covered for COL25A1 between the examined brain regions. However, COL25A1 burden trended higher in the occipital cortex and hippocampus compared to the striatum and cerebellum in cases with intermediate and high ADNC (Fig. 4).

In contrast, the SPOCK3 burden was significantly higher in the hippocampus compared to other brain regions in cases with high ADNC. No significant differences in SPOCK3 deposition were observed between occipital cortex, striatum and cerebellum in cases with high ADNC. In cases with intermediate ADNC, there was no significant difference in SPOCK accumulation among the examined brain regions (Fig. 4).

**Figure 5:**
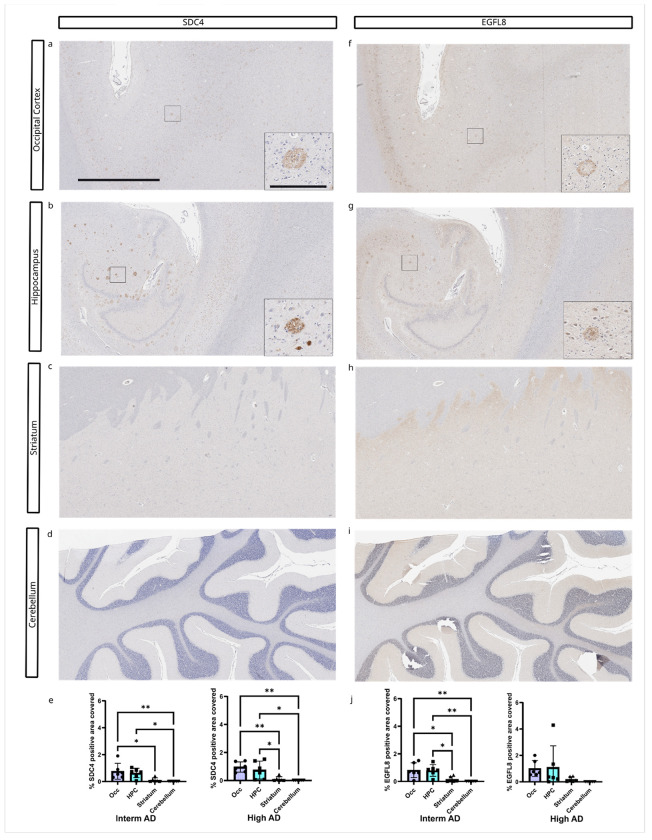
Brain region-specific accumulation of SDC4 and EGFL8. A-D) SDC4 pathology in different brain regions from consecutive sections of a high AD case (sample 16). E) SDC4 pathology burden was significantly higher in the occipital cortex and hippocampus compared to the striatum and cerebellum. F-I) EGFL8 pathology in different brain regions. J) EGFL8 pathology burden was significantly higher in the occipital cortex and hippocampus compared to the striatum and cerebellum in Interm AD cases. Sample size: Interm AD (n=6) and High AD (n=6). Scale bar = 2 mm for the main figure and 200 μm for insets.

SDC4 accumulation was significantly higher in the occipital cortex and hippocampus compared to the striatum and cerebellum (Fig. 5) and EGFL8 load was significantly higher in the hippocampus and occipital cortex in cases with intermediate ADNC, while there were no brain region specific differences in cases with high ADNC (Fig. 5).

#### Matrisome proteins co-accumulate with a subset of Aβ plaques and show distinct localization within Aβ deposits

**Figure 6:**
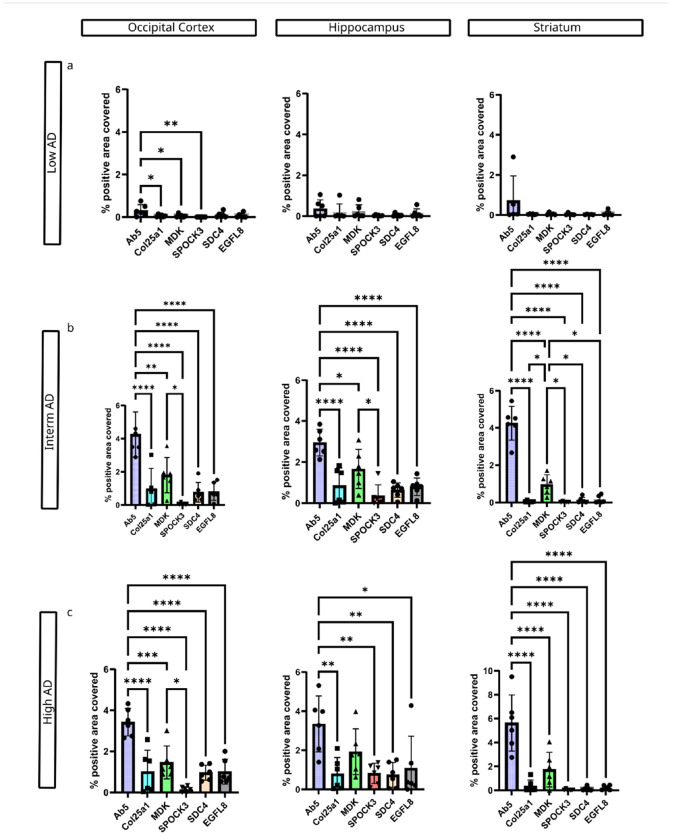
Matrisome proteins stain a subset of Aβ plaques. A) Relative abundance of Aβ plaques and matrisome proteins in Low AD cases across different brain regions. In Low AD cases, Aβ plaque pathology was significantly higher in the occipital cortex compared to other matrisome proteins. No significant differences in the relative abundance of matrisome proteins were observed in the hippocampus or striatum. B) Relative abundance of Aβ plaques and matrisome proteins in Interm AD cases across different brain regions. In Interm AD cases, Aβ plaque burden was significantly higher in the occipital cortex, hippocampus, and striatum compared to matrisome proteins. Among matrisome proteins, MDK burden was significantly higher than SPOCK3 in the hippocampus, higher than COL25A1, SPOCK3, SDC4, and EGFL8 in the striatum. C) Relative abundance of Aβ plaques and matrisome proteins in High AD cases. Similar Interm AD, Aβ plaque burden was significantly higher than matrisome proteins in all brain regions examined. Among matrisome proteins, MDK burden was higher than SPOCK3 in the occipital cortex. Quantification was not performed in the cerebellum due to a lack of staining. Sample size: Low AD (n=6), Interm AD (n=6) and High AD (n=6).

Next, we examined the relative abundance of each matrisome protein in relation to Aβ plaques in our cohort. In cases with low ADNC, Aβ plaque pathology was minimal in all brain regions. Interestingly, SPOCK3, COL25A1, MDK, SDC4, EGFL8 accumulation was also observed in some cases with low ADNC, although at much less abundance compared to Aβ plaques (Fig. 6). In cases with intermediate and high ADNC, the Aβ plaque burden covered approximately 3–6% of the total area (Fig. 6). All matrisome proteins examined here showed significantly less area coverage compared to Aβ plaques. MDK was the most abundant AAP, covering roughly 2% of total area, followed by COL25A1, SDC4 and EGFL8 with less than 1% area covered in the occipital cortex and hippocampus (Fig. 6). SPOCK3 pathology was minimal in all brain regions except the hippocampus, where SPOCK3 burden was comparable to COL25A1 and SDC4 in cases with high ADNC.

Next, we evaluated the extent of overlap and colocalization of matrisome proteins with Aβ plaques using double immunofluorescence labeling. To do this, we co-stained matrisome proteins with an Aβ antibody (Ab5) in cases with high ADNC. Hippocampus proper and fusiform gyrus/inferior temporal gyrus (FG/ITG) were analyzed separately. Qualitatively, we observed that MDK tends to stain the dense core portion of Aβ plaques, while COL25A1 often stains the more diffuse, peripheral portion of Aβ plaques (Supplementary Fig. 2). SPOCK3 stains mostly dystrophic neurites surrounding Aβ plaques. SDC4 and EGFL8 uniformly stain Aβ plaques without preferentially staining the dense core or peripheral portions of Aβ plaques (Supplementary Fig. 2).

**Figure 7:**
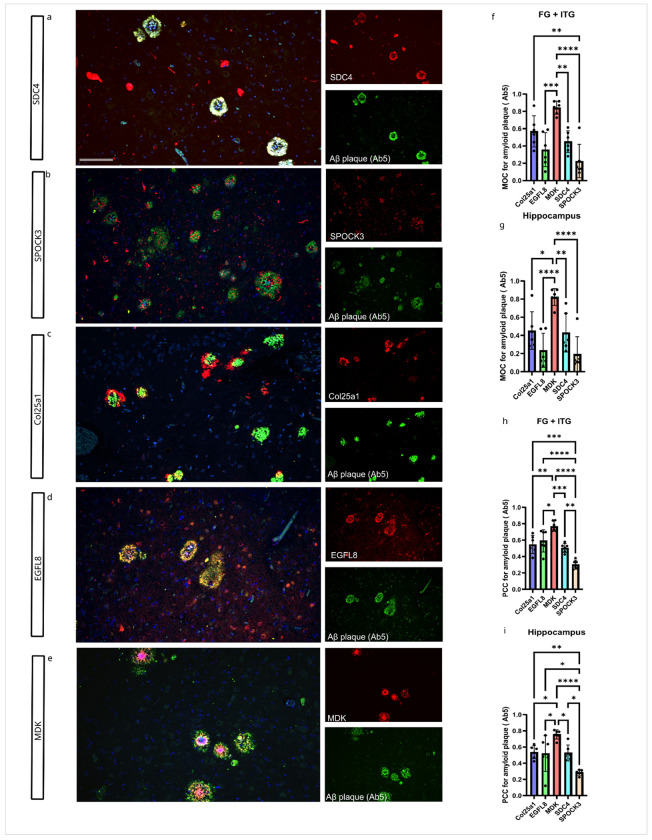
Matrisome proteins colocalized with a subset of Aβ plaques in High AD cases. A-E) Colocalization of SDC4, SPOCK3, COL25A1, EGFL8, MDK with Aβ plaques. F-G) Manders’ overlap coefficient (MOC) of matrisome proteins with Aβ plaques (Ab5) in the FG+ITG and hippocampus. G-H) Pearson’s correlation coefficient (PCC) of matrisome proteins with Aβ plaques (Ab5) in the FG+ITG and hippocampus. Sample size: High AD (n=5). Scale bar = 100 μm

For quantitative assessment of overlap between the AAP staining and Aβ staining, we measured the Pearson’s correlation coefficient (PCC) and thresholded Manders’ overlap coefficient (MOC) using the BIOP JACoP plugin in ImageJ software. MOC, which quantifies the overlapping fraction of colocalized fluorescent signals, showed that over 80% of MDK, over 40% of COL25A1, around 40% of SDC4, over 20% of EGFL8, and around 20% of SPOCK3 co-occurred within Aβ plaques in cases with high ADNC in both hippocampus proper and FG + ITG (Fig. 7f–g). Similarly, PCC, which measures the linear relationship between fluorescent intensities, indicated that MDK showed the strongest correlation with Aβ plaques (70–80%), while COL25A1, EGFL8, and SDC4 each correlated with approximately 50% of the Aβ plaques. In contrast, SPOCK3 showed the weakest correlation with Aβ plaques (Fig. 7h–i).

Manual quantification of Aβ plaques positive for select matrisome proteins in the same cohort showed that SDC4 overlapped with 30–40% of Aβ plaques, SPOCK3 overlapped with 50–60%, and COL25A1, EGFL8, and MDK each overlapped with 60–80% of Aβ plaques (Supplementary Fig. 1d).

### Some matrisome proteins co-localized with tau pathology

**Figure 8:**
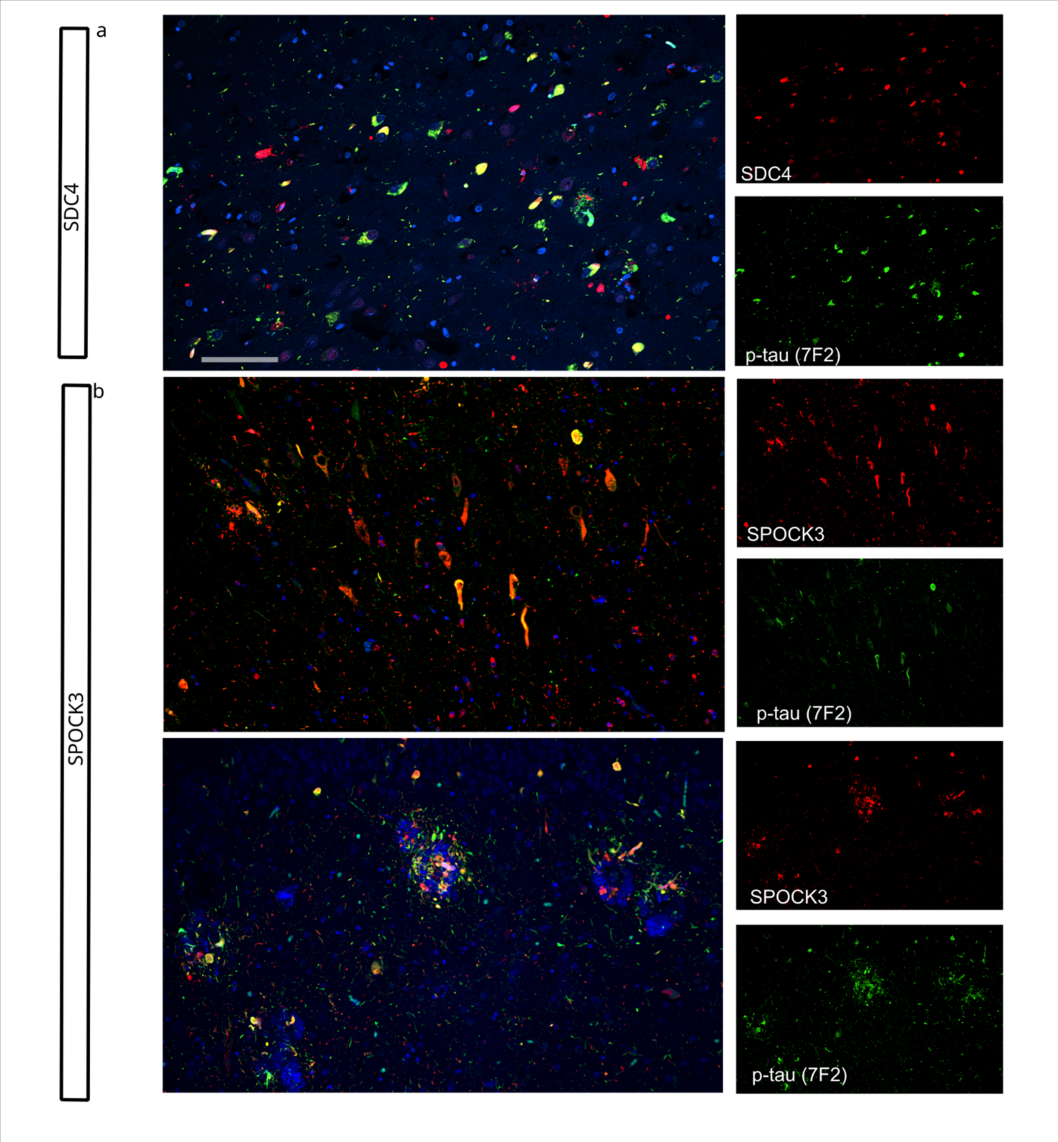
Two matrisome proteins colocalized with subsets of tau pathology in High AD cases. A) SDC4 colocalization with neuronal tau aggregates. B) SPOCK3 colocalization with neuronal tau aggregates and DN. Scale bar = 100 μm

Finally, we examined the colocalization of matrisome proteins with tau pathology. For this analysis, we co-stained hippocampal sections for matrisome proteins and p-tau (7F2)[72]. Out of 5 matrisome proteins we evaluated, only SDC4 and SPOCK3 colocalized with p-tau. SDC4 colocalized with neuronal tau aggregates (Fig. 8a). While SPOCK3 is colocalized with both neuronal tau aggregates and dystrophic neurites (Fig. 8b).

## Discussion

Proteomic studies have shown that many matrisome proteins are upregulated in the brain, CSF, and plasma of AD patients and mouse models of disease[2, 14, 17, 34, 35, 41]. To put the proteomic and transcriptomic changes in the context of pathology, we used in-house-generated antibodies against select matrisome proteins to quantify their accumulation across different stages of the disease and in different brain regions. We found that all examined matrisome proteins accumulated during the progression of ADNC in the occipital cortex and hippocampus, similar to what we reported previously for the frontal cortex[41]. Additionally, we observed brain region-specific differences in the accumulation pattern of select AAP. For instance, MDK co-accumulates with Aβ plaques in the occipital cortex, hippocampus, striatum, and cerebellum, whereas SPOCK3, COL25A1, EGFL8, and SDC4 only show substantial co-accumulation with Aβ plaques in the occipital cortex and hippocampus. SPOCK3 accumulation was significantly higher in the hippocampus compared to other brain regions in cases with high ADNC. Furthermore, colocalization analysis showed that each matrisome protein overlapped Aβ plaque pathology to different degrees and exhibited distinct qualitative staining patterns.

The Amyloid Cascade Hypothesis (ACH) posits that Aβ aggregation and accumulation serve as the trigger/driver of downstream AD pathogenesis. An imbalance in Aβ production (as in familial AD) and Aβ clearance (as in sporadic AD) leads to Aβ aggregation in form of plaques[22, 59]. Many matrisome proteins have heparin sulfate (HS) and heparin sulfate proteoglycan (HSPGs)-binding properties. HSPGs are known to modulate Aβ deposition and serve as reservoirs for Aβ accumulation [32, 45, 47, 57, 65, 71]. Depletion of neuronal HS reduced Aβ deposition in the APP/PS1 mouse model by enhancing the Aβ clearance mechanisms without affecting APP processing and Aβ production [45]. It is likely that a subset of matrisome proteins with HS/HSPG binding properties hinder Aβ clearance by co-accumulating with Aβ plaques. For example, COL25A1- a neuronal type II transmembrane protein- binds and assembles with Aβ fibrils to form a protease resistant aggregate[64]. Overexpression of COL25A1 in the brain of APP transgenic mice remodeled Aβ plaque pathology by compacting Aβ plaques and reducing diffuse plaques[24]. Additionally, overexpression of MDK and PTN increased both Aβ plaque deposition and CAA levels [41]. These findings reveal that certain matrisome proteins can remodel Aβ plaques and promote Aβ deposition by reducing Aβ clearance mechanisms. Blocking the interaction between Aβ plaques and matrisome proteins could potentially enhance Aβ clearance and reduce Aβ plaque deposition.

Aβ deposition in AD is characterized by different morphological subtypes of Aβ plaques. Diffuse plaques, often abundant in elderly individuals without severe cognitive decline, are less neurotoxic, while NP are more associated with neuroinflammation and cognitive decline in AD [70]. In our recent studies, we showed that Gallyas positive NP are significantly associated with microglial and astrocytic clustering compared to non-neuritic diffuse plaques [69]. Differential glial responses to different Aβ plaque subtypes might indicate that Aβ itself is not likely direct neurotoxic, and other factors may be responsible for neurotoxicity. We recently proposed the “amyloid scaffold” hypothesis positing that Aβ plaques scaffold the accumulation of matrisome proteins, which may modulate Aβ toxicity and downstream neurodegeneration[41]. In this scenario, Aβ accumulation is necessary but insufficient to induce downstream neurodegeneration without other factors such as matrisome proteins that co-accumulate within Aβ plaques. Our colocalization study showed that matrisome proteins overlap and colocalize with subset of Aβ plaques. In light of this, it will be important to characterize the microenvironment around Aβ plaques in correlation with these associated matrisome proteins.

Dystrophic neurites associated with Aβ plaques are closely linked with neuroinflammation and neurotoxicity[69]. Interestingly, we show here that SPOCK3 accumulation overlaps substantially with dystrophic neurites. SPOCK3 is a calcium-binding proteoglycan expressed by oligodendrocytes and neurons. Ablation of SPOCK3 expression in mice resulted in thinning of the corpus callosum and fasciculation of cortical fibers[73]. However, SPOCK3 mutant mice were viable and did not exhibit abnormal phenotypes[23]. Proteomic studies using postmortem brain tissue showed that SPOCK3 may link APOE4 to tau pathology [56]. We observed that SPOCK3 pathology burden was highest in the hippocampus. It is intriguing why SPOCK3 did not label dystrophic neurites and tau pathology in the occipital cortex to the same extent as in the hippocampus, considering that the Aβ plaque burden is comparable between the hippocampus and occipital cortex cases with high ADNC. One possible explanation is that the hippocampus is one of the earliest and most severely affected regions in AD, while the occipital cortex is only affected in late stages of ADNC progression. Another possibility is that SPOCK3 expression shows regional differences between hippocampus and occipital cortex. Further studies are warranted to understand why SPOCK3 accumulation is most pronounced in the hippocampus and whether it is associated with local neurotoxicity.

The interaction between Aβ and tau pathology is of great interest in the field. Aβ plaque pathology originates in the neocortex [66], while early tau pathology is observed in the hippocampus[9, 10]. This spatiotemporal disconnect between the two pathological hallmarks raises many questions. In human postmortem brain tissues, we have shown that total Aβ plaque load plateaued in cases with intermediate ADNC. However, the quality of Aβ plaques shifts from Non-NP into NP during progression from intermediate to high ADNC. This shift in Aβ plaque subtypes is associated with the emergence of cortical tau pathology [68]. The spread of tau pathology from the medial temporal lobe into neocortical regions is mediated and facilitated by Aβ plaques and strongly associated with the cognitive decline observed in AD[6, 52, 53]. Recent studies have shown that protective variants in ECM-related genes such as reelin, APOE, and fibronectin delay disease onset and are associated with reduced tau pathology in the entorhinal cortex and frontal cortex [7, 13, 46], suggestive of ECM components mediating Aβ-related tau pathology. A study from the Holtzman lab showed that injecting of AD-brain-derived tau extracts into humanized APOE3 Christchurch knock-in mice crossed with the Aβ plaque-depositing APPPS1 model showed reduced NP tau compared to APOE3 expressing mice crossed with APPS1 mice[13]. Similarly, human cell culture studies mimicking the protective APOE Christchurch variant, also demonstrated reduced Aβ-mediated tau pathology[13, 51]. Those studies speculated that the weak binding of APOE Christchurch to HSPGs may regulate the reduced spread and seeding of tau. SDC4 is a cell-surface HSPG with altered expression levels in AD. Immunohistochemistry showed that SDC4 is associated with Aβ plaque and tau pathology[40, 47]. Notably, SDC4 has been shown to be associated with the internalization of Aβ and tau species in cell culture assays [40, 44]. In our study, we observed that SDC4 co-accumulates with both Aβ plaques and tau pathology, but SDC4 staining of tau pathology was relatively scarce. Both SDC4 and SPOCK3 are secretory proteins with HSPG-binding properties. Co-accumulation of SDC4 and SPOCK3 with tau pathology may suggest that these two proteins are involved in the spread and seeding of tau. However, SDC4 and SPOCK3 have not been identified in tau interactome studies[18, 38, 67]. Future studies could explore how these ECM proteins might mediate tau seeding and spread.

The brain region-specific accumulation of matrisome proteins we observed in our studies also warrants further discussion. Co-deposition of MDK was noted in all brain regions while SPOCK3, COL25A1, EGFL8, and SDC4 were largely observed in the occipital cortex and hippocampus. The region-specific accumulation of matrisome proteins can be correlated with regional vulnerability and susceptibility to AD and the distribution of Aβ plaque subtypes. The hippocampus and the occipital cortex are more severely affected by ADNC, while the striatum and cerebellum are mostly exhibiting diffuse Aβ pathology[33]. The absence of SPOCK3, COL25A1, EGFL8, and SDC4 accumulation in the striatum and cerebellum likely indicates that these matrisome proteins largely co-accumulate with Aβ plaque subtypes such as dense-cored plaque or NP, rather than diffuse plaques. Alternatively, it is possible that certain ECM proteins, including SPOCK3, COL25A1, EGFL8, and SDC4, are required for the formation of more fibrillar Aβ plaques. In the absence of co-deposition of those proteins in the cerebellum, only diffuse plaques develop. Heparan sulfate proteoglycans have been shown to be present in diffuse plaques in the hippocampus but not in those in the cerebellum[63]. We have observed COL25A1, SDC4, and EGFL8 staining in diffuse plaque in the occipital cortex and hippocampus. However, this staining was not observed in diffuse plaques within the striatum and cerebellum.

More importantly, there is a growing need for early biomarkers for both the diagnosis and treatment of AD. For a long time, AD has been diagnosed only after clinical symptoms manifest in the later stage of the disease. However, immunotherapy studies in clinical trials have revealed that treating AD patients after the onset of clinical symptoms is largely ineffective and often too late during disease progression. Recently there has been increasing consensus among researchers to define AD as a biological disease based on biomarker changes rather than solely on clinical symptoms and postmortem pathology[30, 31, 39, 60]. This shift in diagnostic criteria underscores the urgent need to develop early biomarkers. SMOC1, a matrisome protein, has been shown to increase in the earliest preclinical stages of AD in human brain tissue and CSF[3, 11, 26, 36]. Other matrisome proteins may also serve as early biomarkers and should be investigated in both plasma and CSF. In the brain, we showed that some matrisome proteins colocalized more tightly with Aβ plaques (MDK), while other matrisome proteins loosely colocalized with Aβ plaques (SPOCK3).

In conclusion, our study demonstrates the disease stage and brain region-specific accumulation of several matrisome proteins and highlights the colocalization of AAP with AD pathological hallmarks. How these proteins affect AD pathophysiology including Aβ and CAA deposition, tau seeding and spread, and conversion of Aβ plaque subtypes needs to be validated future studies.

## Figures and Tables

**Table 1:** Neuropathology data for cases used in this study

Sample	Neuropath	Thal	Braak	CERAD	APOE	Sex	Age	PMI	MMSE score	alpha synuclein	LATE-NC
1	Low AD	2	II	none	3/4	F	63	168	NA	No	No
2	Low AD	1	III	none	3/4	F	79	72	NA	No	No
3	Low AD	3	II	sparse	3/3	M	79	34	NA	No	No
4	Low AD	3	II	sparse	3/3	M	81	144	NA	No	No
5	Low AD	4	0	none	3/4	F	82	22	20/30	No	No
6	Low AD	1	III	none	3/3	F	92	8	NA	No	No
7	Intermediate AD	5	IV	frequent	3/3	M	72	72	NA	No	No
8	Intermediate AD	5	III	sparse	2/4	M	78	4	18/30*	No	No
9	Intermediate AD	3	VI	moderate	3/3	M	82	13	1/30	No	No
10	Intermediate AD	5	III	moderate	3/3	M	83	22	NA	No	No
11	Intermediate AD	4	IV	frequent	3/3	F	89	8	24/30	No	No
12	Intermediate AD	5	IV	frequent	3/3	F	100	18	NA	No	No
13	High AD Pure	5	VI	frequent	4/4	M	70	8	NA	No	No
14	High AD Pure	4	V	frequent	3/4	M	77	5	21/30	No	No
15	High AD Pure	5	V	frequent	3/3	F	78	5	NA	No	No
16	High AD Pure	5	VI	frequent	3/3	M	79	21	16/30	No	No
17	High AD Pure	5	VI	frequent	3/3	M	83	7	NA	No	No
18	High AD Pure	4	V	frequent	3/4	F	85	18	23/30	No	No

* MOCA score was converted into MMSE score

## Data Availability

The data that support the findings of this study are available upon request.
